# Medical Law Suit

**Published:** 1857-02

**Authors:** 


*Medical Law Suit.*—In the matter of John D. Hill *vs.* The Medical Society of the County of Erie, we learn that the lawyers are busily engaged upon complaints and answers, rejoinders and rebutters. The case is shaping in such a manner as to test the powers of county societies; and will be one of great interest to the profession at large. From the character of the court before which it is brought, and the eminence of the legal gentlemen employed, we anticipate that the case will go far to settle many mooted points of medical law.

